# A Rare Finding of Mosaic 45,XX,der(13;21)(q10;q10)[15]/46,XX,r(13)(p11.2q33) Following Abnormal Prenatal Chromosomal Microarray Testing

**DOI:** 10.1155/crig/7026115

**Published:** 2026-08-31

**Authors:** Katherine M. Haines, Deborah M. Hazard, Billie Carstens, Peter M. Brzeskiewicz, Thomas M. Gilfillan, Hala Nijmeh, Chandra Perez-Gill, Mikayla Stoecker, Farrah Rajabi, Mary M. Haag, Stephen J. Wicks

**Affiliations:** ^1^ Colorado Genetics Laboratory, Department of Pathology, Anschutz, University of Colorado Anschutz, Colorado, USA, ucdenver.edu; ^2^ Section of Genetics and Metabolism, Department of Pediatrics, University of Colorado Anschutz, Aurora, Colorado, USA, ucdenver.edu; ^3^ Children’s Hospital Colorado, Aurora, Colorado, USA, childrenscolorado.org

## Abstract

Mosaicism for both a ring chromosome and Robertsonian translocation is a rarely reported cytogenetic phenomenon. We describe a case referred for testing following abnormal noninvasive prenatal screening for trisomy 13. Prenatal chromosomal microarray (CMA) testing identified a mosaic 3.6‐Mb terminal loss in 13q34, which was confirmed by postnatal CMA. At birth, the phenotype included a small head circumference (3rd percentile) and a patent ductus arteriosus. Conventional chromosome analysis identified mosaicism of two abnormal cell lines, one containing der(13;21)(q10;q10) and the other containing r(13)(p11.2q33). This case demonstrates the utility of conventional chromosomal analysis following an abnormal CMA result.

## 1. Introduction

Diagnostic prenatal chromosomal microarray (CMA) is recommended as follow‐up testing for individuals with positive prenatal cell‐free DNA (cfDNA) screening results [1]. CMA detects gains and losses in DNA copy number with a resolution of approximately five kilobases. However, CMA does not detect the structural alterations underlying copy number changes and does not detect balanced chromosomal rearrangements or regions with limited resolution due to repetitive genomic sequences or poor probe coverage. Here, we present the cytogenetic findings of an individual with mosaic 13q34 loss identified on prenatal CMA and mosaic der(13;21)(q10;q10)/r(13)(p11.2q33) identified on follow‐up postnatal chromosome analysis.

## 2. Case Presentation

A newborn female infant was referred because of abnormal prenatal cfDNA screening results. Prenatal cfDNA screening by a DNA sequencing–based methodology was positive for increased risk of trisomy 13. Confirmatory CMA performed at an outside laboratory on amniotic fluid identified mosaic loss of the q (long) terminal end of chromosome 13, 13q34(111,549,983‐115,107,733)x1∼2. Prenatal aneuploidy FISH detection studies performed on the same specimen were normal for chromosomes 13, 18, 21, and X/Y. At birth, the individual’s phenotype included a small head circumference (32 cm, 3^rd^ percentile) with weight in the 26^th^ percentile and height in the 33^rd^ percentile. Additionally, mild widely spaced eyes with epicanthal folds, upslanted palpebral fissures, and 5^th^ finger clinodactyly were noted. Initial echocardiogram demonstrated a patent ductus arteriosus (PDA) and increased aortic size (Z‐score +2.22). Initial hearing screening was normal, and cytomegalovirus testing as a cause for the small head circumference was negative.

**FIGURE 1:**
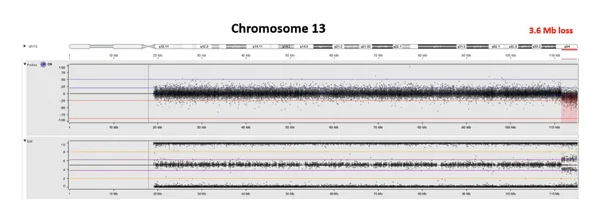
Chromosomal microarray analysis of peripheral blood detected a 3.6‐MB loss at the terminal region of chromosome 13 (in 13q34).

Postnatal CMA (Illumina Infinium Global Diversity Bead array, GRCh37) performed at the Colorado Genetics Laboratory confirmed the mosaic 3.6‐Mb loss in 13q34, arr[GRCh37] 13q34(111545554_115106996)x1 ∼ 2 although minor differences in the reported breakpoints likely reflect the use of different microarray platforms. An additional 308‐kb loss in 16p13.3, 16p13.3(6780311_7088336)x1, was also detected. The 16p13.3 loss did not contain any OMIM, Online Mendelian Inheritance in Man, genes and was found to be inherited from the unaffected mother by parental targeted CMA testing. The mosaic 13q34 loss was found to be *de novo* and contained six OMIM genes: *ATP11A*, *F7*, *F10*, *PROZ*, *GRK1*, and *CHAMP1* (Figure 1). Heterozygous pathogenic variants in *CHAMP1* are associated with autosomal dominant neurodevelopmental disorder with hypotonia, impaired language, and dysmorphic features [2]. Individuals with microdeletions in this region are reported to present with a milder clinical phenotype, including microcephaly and cardiac findings [3]. Heterozygous pathogenic variants in *ATP11A* are associated with autosomal dominant deafness, with onset, typically postlingual, during the first or second decade of life [4].

**FIGURE 2:**
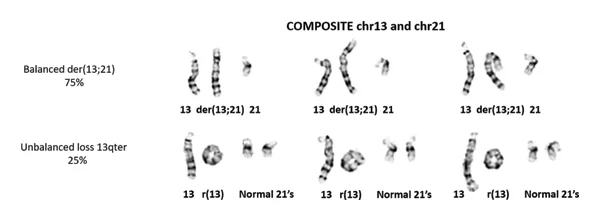
Composite image of chromosome analysis of peripheral blood. (A) 75% of the cells analyzed contained one normal chromosome 13, one normal chromosome 21, and a Robertsonian translocation, der(13;21)(q10;q10). (B) 25% of the cells analyzed contained one normal chromosome 13, two normal chromosome 21s, and a ring chromosome 13.

Routine chromosome analysis of mitogen‐stimulated peripheral blood lymphocytes was performed for the individual and parents. The individual’s analysis revealed mosaicism for two abnormal cell lines. Seventy‐five percent of the cells analyzed contained a Robertsonian translocation, der(13;21)(q10;q10), and one normal chromosome 13 (Figure 2A). Twenty‐five percent of the cells analyzed contained a ring chromosome 13, r(13)(p11.2q33), and one normal chromosome 13 (Figure 2B). The percentage of cells containing the r(13)(p11.2q33) abnormality corresponded to the proportion of mosaic terminal 13q34 deletion estimated by CMA results. No normal 46,XX cell line was detected and both abnormalities were determined to be *de novo*. Chromosome analysis was normal for both parents, with no balanced der(13;21)(q10;q10) identified.

At five months of age, the individual demonstrated resolution of the PDA and stable mild ascending aortic dilation (Z‐score +2.4). At 10 months of age, the ascending aorta continued to be mildly dilated (Z‐score +3.2). The individual’s growth was overall small with a head circumference continuing to track just below the 3^rd^ percentile (Z‐score −1.99) at 13 months of age, with height and weight both tracking at the 5^th^ percentile. At 18 months of age, the individual was developing appropriately for age with no significant developmental concerns reported. Recommendations for the individual’s ongoing care included developmental support with early intervention, yearly hearing screening, and ongoing cardiology review.

## 3. Discussion

This case illustrates the utility of conventional chromosome analysis following an abnormal CMA result. CMA detects only gains and losses in DNA copy number and does not have the ability to detect rearrangements and copy number changes in highly repetitive regions such as the p (short) arms of acrocentric chromosomes [5]. In the present case, prenatal and postnatal CMA detected only the mosaic loss in 13q34. Although the loss in 13q34 likely explains the individual’s cardiac findings, the presence of the Robertsonian translocation, der(13;21)(q10;q10), has additional future reproductive implications for this individual because carriers of Robertsonian translocations have an increased risk of a conceptus with an unbalanced chromosome complement. Mosaicism involving both a ring chromosome and a Robertsonian translocation has been reported only rarely. In previously reported cases, the Robertsonian translocation involved two homologous chromosomes. These included mosaic r(13) and der(13;13)(q10;q10) [6–9], r(14) and der(14;14)(q10;10) [10, 11], and r(21) and der(21;21)(q10;10) [11, 12]. Mosaic isochromosome 18, iso psu dic(18)(p11), with r(18) has also been reported [13]. Notably, a subset of these individuals were also mosaic for a normal 46,XX or 46,XY cell line.

A mechanism accounting for the common origin of a ring chromosome and a translocated chromosome has been proposed by others. This mechanism requires three strand breaks during an early mitosis, with two breaks at homologous sites in the p arm of both sister chromatids and a third break at the distal end of the q arm of one sister chromatid [10, 13]. In this mechanism, the chromatid with both a p and q arm break forms a ring following chromatid separation at anaphase. The chromatid with the single break in the p arm then duplicates during the S phase and forms a union between the two broken p arm ends, resulting in a translocation.

**FIGURE 3:**
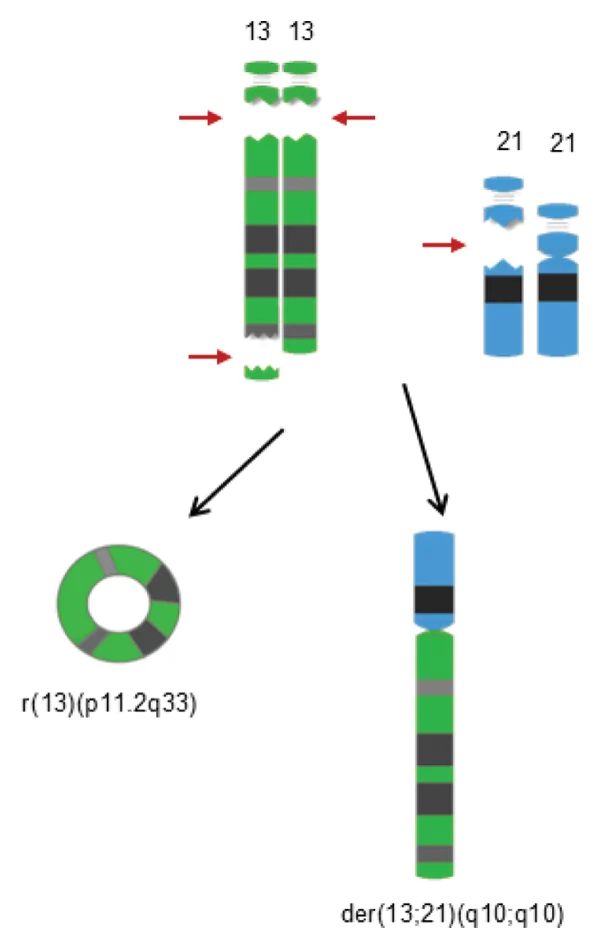
Proposed mechanism for common origin of the r(13)(p11.2q33) and der(13;21)(q10;q10). Following replication during an early mitosis, four total strand breaks (three breaks involving sister chromatid 13 and one break involving sister chromatid 21) are required.

In the present case, we hypothesize through the proposed mechanism, chromosome 13 acquired three breaks. However, to form a Robertsonian translocation between two nonhomologous chromosomes, a fourth break in the p arm of chromosome 21 must also have occurred, with the (13;21) translocation formed after anaphase but before the next S phase replication (Figure 3).

The phenotypic consequences of mosaicism involving both a ring chromosome and a Robertsonian translocation are highly variable and depend on the extent of monosomy or trisomy resulting from these rearrangements. In many of the reported individuals, the level of mosaicism was found to be variable between peripheral blood and fibroblasts, with differences reported between fibroblasts sampled from different skin locations [10]. Additionally, mosaicism of the number and size of ring chromosomes was observed [13]. Studies have also shown that the distribution of the ring chromosome and Robertsonian translocation changes over time in tissue culture, with selective pressure favoring cells with a balanced chromosomal content [14]. It is important to consider in the present case that only peripheral blood was examined, and levels of mosaicism may differ depending on the tested specimen. Thus, the development of this individual’s phenotype may be affected by higher proportions of the ring chromosome 13 abnormality in certain tissues and a subsequent higher proportion of loss of the genes in 13q34. There are also likely reproductive implications for this individual, as the chromosomal complement in gonadal tissue cannot be determined from peripheral blood analysis.

The most common phenotype associated with ring autosomes, independent of the chromosome involved, is growth failure with few or no minor anomalies. Individuals with ring autosomes have been reported with a small head circumference that is proportional to the generalized growth failure [15]. Microcephaly and short stature have both been reported in 13q33–q34 deletion syndromes, but the reported frequency varies across cohorts [3, 16]. Given this phenotypic overlap, it remains uncertain whether this individual’s small size and head circumference are attributable to the ring chromosome itself or to loss of 13q34 material resulting from ring formation. Cardiac anomalies have been reported in a subset of individuals with 13q33‐13q34 deletions, including Tetralogy of Fallot, atrial septal defects, and ventricular septal defects [3, 16, 17]. The PDA and increased aortic size observed in this individual have not been previously reported in association with this deletion. Additional phenotypes associated with deletions affecting 13q34 are variable and dependent on the gene content, though neurodevelopmental phenotypes associated with the loss of *CHAMP1* are reported in the majority of cases [16]. Importantly, all reported 13q34 deletions have been heterozygous. The present case involves a mosaic 13q34 deletion detected at approximately 25 percent in peripheral blood, and the level of mosaicism may differ among tissues. Because tissue‐specific mosaicism can influence phenotypic expression, the degree of expected overlap with individuals carrying heterozygous deletions cannot be predicted.

Interpretation of this mosaic finding requires consideration of the methodology used and the specimen analyzed by each testing modality, as both can influence the detection and quantification of mosaicism. cfDNA screening assays rely on the detection of DNA fragments originating from placental trophoblasts and are most representative of the genetic complement of the placenta, which may differ from that of the fetus in the case of confined placental mosaicism [18, 19]. Additionally, cfDNA assays are not typically designed to detect mosaicism. The differences in methodology between diagnostic assays (i.e., CMA and karyotyping) and cfDNA screening may explain the discordant cfDNA result of trisomy 13 in this case; however, a true difference between the placental and fetal DNA complements cannot be ruled out. In the context of an abnormal cfDNA finding, follow‐up diagnostic testing is required. In the case presented, diagnostic prenatal testing was performed on an amniotic fluid specimen. While the DNA found in amniotic fluid is primarily fetal derived, levels of mosaicism detected in this specimen type may poorly reflect the distribution of mosaicism in other tissues, including peripheral blood. Thus, confirmation of the mosaic prenatal finding in a postnatal specimen was warranted and testing to evaluate the level of mosaicism in different tissues may be considered in the future. In conclusion, we report mosaic chromosome 13 abnormalities 45,XX,der(13;21)(q10;q10)[15]/46,XX,r(13)(p11.2q33)[5] in a newborn female infant with small head circumference and cardiac findings. This report highlights the importance of conventional chromosome analysis in fully characterizing chromosomal copy number changes detected by CMA. We also hypothesize a novel mechanism to account for a common origin of both the r(13) and der(13; 21)(q10;q10) chromosome abnormalities.

## Author Contributions

Katherine M. Haines drafted the original manuscript. Farrah Rajabi, Mary M. Haag, and Stephen J. Wicks contributed to editing the manuscript. Billie Carstens and Katherine M. Haines generated the figures. Peter M. Brzeskiewicz, Deborah M. Hazard, Thomas M. Gilfillan, and Hala M. Nijmeh performed clinical analysis of karyotype and CMA results for this case. Mikayla Stoecker and Chandra Perez‐Gill were responsible for report interpretation. Farrah Rajabi is involved in the patient’s clinical care.

## Funding

The authors did not receive any funding for this research.

## Consent

Written informed consent was obtained from the parents of the patient for publication of this case report.

## Conflicts of Interest

The authors declare no conflicts of interest.

## Data Availability

Data sharing is not applicable to this article as no datasets were generated or analyzed during the current study.

## References

[1] Cherry A. M. , Akkari Y. M. , Barr K. M. et al., Diagnostic Cytogenetic Testing Following Positive Noninvasive Prenatal Screening Results: A Clinical Laboratory Practice Resource of the American College of Medical Genetics and Genomics (ACMG), Genetics in Medicine. (2017) 19, no. 8, 845–850, 10.1038/gim.2017.91. 28726804

[2] Deciphering Developmental Disorders S. , Large-Scale Discovery of Novel Genetic Causes of Developmental Disorders, Nature. (2015) 519, no. 7542, 223–228, 10.1038/nature14135. 25533962 PMC5955210

[3] Levy T. , Pichardo T. , Silver H. et al., Prospective Phenotyping of CHAMP1 Disorder Indicates That Coding Mutations may Not Act Through Haploinsufficiency, Human Genetics. (2023) 142, no. 9, 1385–1394, 10.1007/s00439-023-02578-6. 37454340 PMC10449971

[4] Pater J. A. , Penney C. , O’Rielly D. D. et al., Autosomal Dominant Non-Syndromic Hearing Loss Maps to DFNA33 (13Q34) and Co-Segregates With Splice and Frameshift Variants in ATP11A, a Phospholipid Flippase Gene, Human Genetics. (2022) 141, no. 3-4, 431–444, 10.1007/s00439-022-02444-x. 35278131 PMC9035003

[5] Jarmuz-Szymczak M. , Janiszewska J. , Szyfter K. , and Shaffer L. G. , Narrowing the Localization of the Region Breakpoint in Most Frequent Robertsonian Translocations, Chromosome Research. (2014) 22, no. 4, 517–532, 10.1007/s10577-014-9439-3. 25179263 PMC4257996

[6] Gentile M. , Buonadonna A. L. , Cariola F. , Fiorente P. , Valenzano M. C. , and Guanti G. , Molecular and Cytogenetic Characterisation of an Unusual Case of Partial Trisomy/Partial Monosomy 13 Mosaicism: 46,Xx,R(13)(P11Q14)/46,Xx,Der(13)T(13;13)(Q10;Q14), Journal of Medical Genetics. (1999) 36, no. 1, 77–82, 10.1136/jmg.36.1.77. 9950374 PMC1762957

[7] Duckett D. P. , Porter H. J. , and Young I. D. , Trisomy/Partial Monosomy 13 Mosaicism Associated With Relatively Mild Clinical Malformation, Annales de Genetique. (1992) 35, no. 2, 113–116. 1524408

[8] Fryns J. P. , Kleczkowska A. , Jaeken J. , Van Herck K. , and Van den Berghe M. H. , Mosaic 13 Trisomy due to de Novo 13/13 Translocation With Subsequent Fission, Karyotype. (1989) 46. 2486064

[9] Jalal S. M. , Martin J. A. , Benjamin T. R. , Kukolich M. K. , and Townsend-Parcham J. K. , Unusual Mosaic Trisomy 13 Through 13/13 Translocation and Monosomy 13 With a Small Ring, Annales de Genetique. (1990) 33, no. 3, 173–175. 2288463

[10] Cantu E. S. , Thomas I. T. , and Frias J. L. , Unusual Cytogenetic Mosaicism Involving Chromosome 14 Abnormalities in a Child With an MR/MCA Syndrome and Abnormal Pigmentation, Clinical Genetics. (1989) 36, no. 3, 189–195, 10.1111/j.1399-0004.1989.tb03187.x. 2676269

[11] Pangalos C. , Velissariou V. , Ghica M. , and Liacacos D. , Ring-14 and Trisomy 14q in the Same Child, Annales de Genetique. (1984) 27, no. 1, 38–40. 6609671

[12] Dallapiccola B. , Bianco I. , Brinchi V. et al., t(21q21q)/r[t(21q21q)] Mosaic in Two Unrelated Patients With Mild Stigmata of Down’s Syndrome, Annales de Genetique. (1982) 25, no. 1, 56–58. 6211124

[13] Madan K. , Vlasveld L. , and Barth P. G. , Ring-18 and Isopseudodicentric-18 in the Same Child: A Hypothesis to Account for Common Origin, Annales de Genetique. (1981) 24, no. 1, 12–16. 6971609

[14] Mikkelsen M. and Niebuhr E. , A Ring Chromosome (46,XY,13r) Occurring in a Family With a D-D Translocation 13-14-t(13q 14q), Annales de Genetique. (1969) 12, no. 1, 51–56. 5306712

[15] Kosztolányi G. , Does “Ring Syndrome” Exist? An Analysis of 207 Case Reports on Patients With a Ring Autosome, Human Genetics. (1987) 75, no. 2, 174–179. 3817812 10.1007/BF00591082

[16] Sagi-Dain L. , Goldberg Y. , Peleg A. et al., The Rare 13q33-q34 Microdeletions: Eight New Patients and Review of the Literature, Human Genetics. (2019) 138, no. 10, 1145–1153, 10.1007/s00439-019-02048-y. 31321490

[17] Huang C. , Yang Y. F. , Yin N. et al., Congenital Heart Defect and Mental Retardation in a Patient With a 13q33.1-34 Deletion, Gene. (2012) 498, no. 2, 308–310, 10.1016/j.gene.2012.01.083. 22366306

[18] Levy B. , Hoffmann E. R. , McCoy R. C. , and Grati F. R. , Chromosomal Mosaicism: Origins and Clinical Implications in Preimplantation and Prenatal Diagnosis, Prenatal Diagnosis. (2021) 41, no. 5, 631–641, 10.1002/pd.5931. 33720449 PMC8176867

[19] Grati F. R. , Chromosomal Mosaicism in Human Feto-Placental Development: Implications for Prenatal Diagnosis, Journal of Clinical Medicine. (2014) 3, no. 3, 809–837, 10.3390/jcm3030809. 26237479 PMC4449651

